# Transthyretin-related Familial Amyloid Polyneuropathy (TTR-FAP) caused by a very rare, de novo mutation in a Polish patient.

**DOI:** 10.1186/1750-1172-10-S1-P25

**Published:** 2015-11-02

**Authors:** Marta Lipowska, Dorota Rowczenio, Janet Gilbertson, Philip N Hawkins, Agnieszka Ptasinska-Perkowska, Hanna Drac

**Affiliations:** 1 Medical University of Warsaw, Deparment of Neurology, 02-097, Warsaw, Poland; 2 UCL Division of Medicine, National Amyloidosis Centre, NW3 2PF, London, UK; 3 Medical University of Warsaw, 3. Transplantation Institute, 02-006, Warsaw, Poland

## Introduction

TTR-FAP is rarely diagnosed in Poland – thus far only a few patients of Polish origin have been diagnosed with this type of amyloidosis. We present a case of TTR-FAP in a Polish patient in whom we discovered a rare TTR mutation.

## Materials and methods

56 years old man presented with a 5 -year history of progressive polyneuropathy. His symptoms started with numbness and paresthesia in the feet followed by weakness. Three years after similar symptoms spread into his hands. Subsequently he developed autonomic symptoms: diarrhea, neurogenic bladder with mild urine retention and impotence. Due to progressive neuropathy his movements were limited such that he required rollator for walking after four years of symptoms. Excessive weight loss resulted in cachexia and BMI of 17,5. His family history was negative.

The patient underwent detailed clinical evaluation, since his symptoms were higly suggestive of FAP his blood sample and fat biopsy were sent to the National Amyloidosis Centre (NAC) in London for genetic and histopathological studies.

## Results

Neurography revealed diffuse sensory and motor axonopathy. Cardiomyopathy was reported on MR and ultrasounds, but it was clinically mild. Vitreous opacities were found, with no clinical impact.

Genetic analysis demonstrated that the patient was heterozygous for p.Asp58Val (D38V) TTR mutation. DNA isolated from his parents and brother was negative, indicating de novo mutation. Amyloid deposits were identified on fat biopsy by pathognomonic green birefringence when stained with Congo red and viewed under crossed polarised light. The amyloid stained specifically with antibodies to transthyretin.

## Conclusions

We report a patient with a very rare TTR p.Asp58Val mutation who presented with typical clinical picture of late-onset TTR-FAP with predominant polyneuropathy. Due to advanced stage of disease the patient had no indications for tafamidis or liver transplantation. This variant has previously been identified in a Ghanaian male who presented at similar age with predominant polyneuropathy (Lachmann, 2002). In our patient this mutation has occurred de novo, which is uncommon in TTR-FAP.

